# Single genome analysis reveals genetic characteristics of Neuroadaptation across HIV-1 envelope

**DOI:** 10.1186/s12977-014-0065-0

**Published:** 2014-08-15

**Authors:** Teresa H Evering, Edwin Kamau, Leslie St. Bernard, Charles B Farmer, Xiang-Peng Kong, Martin Markowitz

**Affiliations:** Aaron Diamond AIDS Research Center, an affiliate of the Rockefeller University, New York, USA; Department of Biochemistry and Molecular Pharmacology, NYU School of Medicine, New York, USA; Current address: John’s Hopkins School of Medicine, Baltimore, Maryland USA

**Keywords:** Human immunodeficiency virus (HIV-1), HIV-1 envelope, HIV-associated neurocognitive disorder (HAND), Central nervous system, Viral evolution, Single genome sequencing

## Abstract

**Background:**

The widespread use of highly effective, combination antiretroviral therapy (cART) has led to a significant reduction in the incidence of HIV-associated dementia (HAD). Despite these advances, the prevalence of HIV-1 associated neurocognitive disorders (HANDs) has been estimated at approximately 40%-50%. In the cART era, the majority of this disease burden is represented by asymptomatic neurocognitive impairment and mild neurocognitive disorder (ANI and MND respectively). Although less severe than HAD, these diagnoses carry with them substantial morbidity.

**Results:**

In this cross-sectional study, single genome amplification (SGA) was used to sequence 717 full-length HIV-1 envelope (*env*) clade B variants from the paired cerebrospinal fluid (CSF) and blood plasma samples of fifteen chronically infected HIV-positive individuals with normal neurocognitive performance (NCN), ANI and MND. Various degrees of compartmentalization were found across disease states and history of cART utilization. In individuals with compartmentalized virus, mean HIV-1 *env* population diversity was lower in the CSF than plasma-derived variants. Overall, mean V1V2 loop length was shorter in CSF-derived quasispecies when compared to contemporaneous plasma populations, and this was found to correlate with a lower mean number of N-linked glycosylation sites in this region. A number of discrete amino acid positions that correlate strongly with compartmentalization in the CSF were identified in both variable and constant regions of gp120 as well as in gp41. Correlated mutation analyses further identified that a subset of amino acid residues in these compartmentalization “hot spot” positions were strongly correlated with one another, suggesting they may play an important, definable role in the adaptation of viral variants to the CSF. Analysis of these hot spots in the context of a well-supported crystal structure of HIV-1 gp120 suggests mechanisms through which amino acid differences at the identified residues might contribute to viral compartmentalization in the CSF.

**Conclusions:**

The detailed analyses of SGA-derived full length HIV-1 *env* from subjects with both normal neurocognitive performance and the most common HAND diagnoses in the cART era allow us to identify novel and confirm previously described HIV-1 *env* genetic determinants of neuroadaptation and relate potential motifs to HIV-1 *env* structure and function.

**Electronic supplementary material:**

The online version of this article (doi:10.1186/s12977-014-0065-0) contains supplementary material, which is available to authorized users.

## Background

HIV-1 infection of the central and peripheral nervous systems (CNS, PNS) can result in a wide range of pathological and clinical manifestations. These include HIV-associated encephalopathy, dementia and sensory neuropathies – all of which contribute significantly to morbidity and mortality [1–3]. The widespread use of highly effective combination antiretroviral therapy (cART) has led to a clear reduction in the incidence of HIV-associated dementia (HAD), one of the most severe manifestations of HIV-1 CNS infection [4]. Despite this decrease, HIV-1 associated neurocognitive disorders (HANDs) persist in the cART era [5], with an estimated prevalence of approximately 40-50% [6,7]. Proposed in 2007, current research nosology recognizes three major categories of disease: asymptomatic neurocognitive impairment (ANI), HIV-associated mild neurocognitive disorder (MND), and HAD [8]. Asymptomatic neurocognitive impairment is defined as acquired impairment in at least 2 cognitive ability domains in the absence of criteria for delirium or dementia, with no other preexisting cause in the absence of interference with daily functioning. Mild neurocognitive disorder shares these criteria, with the addition of the demonstration of at least mild interference in daily functioning [8]. When compared to previous criteria that defined only two levels of neurologic manifestations of HIV - HAD and minor cognitive motor disorder (MCMD) - a diagnostic scheme including ANI was found to have improved positive predictive power, sensitivity and specificity when HIV-related brain involvement was defined as the neuropathological diagnosis of HIV encephalitis (HIVE) at autopsy [9]. In addition, HIV-1 associated neurocognitive disorders less severe than frank dementia have been shown to be independently associated with an increased risk for mortality in those with HIV [10] and a recent study suggests the self-report of functional performance may underestimate symptomatic impairment in HAND [11].

In the normal state, anatomic, physiologic, and immunoregulatory mechanisms ensure the immune privilege of the brain, preventing recognition of foreign antigens and diminishing or blocking inflammatory responses [12,13]. HIV enters the CNS during primary infection [14,15]. In the “Trojan horse” hypothesis, HIV is postulated to traffic across the blood-brain barrier (BBB) via the infiltration of infected CD4+ monocytes and perivascular macrophages [16,17]. HIV may also enter the CNS via infected lymphocytes or as cell-free virus [18,19]. A recent study by Schnell et al. identified both T cell-tropic and macrophage-tropic HIV-1 populations genetically compartmentalized to the cerebrospinal fluid (CSF) of individuals with HAD [20]. Irrespective of the method of entry, the infection and activation of monocytes and macrophages are thought to play an important role in the pathogenesis of HIVE as well as HAD [21–23]. Direct neuronal infection of HIV-1 is not believed to occur [13] and indirect mechanisms inducing neuronal signaling and apoptosis are thought to play a major role in disease pathogenesis [22,24].

The HIV-1 envelope (*env*) gene encodes important immune targets and host-range determinants [25]. This major viral protein mediates binding to the CD4 receptor on target cells, undergoes conformational changes that allow for viral entry [26] and has been postulated to play an important role in both neuroinvasion and neurotropism [27]. Genetic differences and phylogenetic compartmentalization of CNS- and blood-derived partial HIV-1 *env* sequences from the same patient have been documented in several studies [28–32]. Similar analyses have been performed using full-length HIV-1 *env* cloned from individuals with end-stage disease [33]. Analysis of clonal sequences from chronically infected individuals have suggested that HIV-1 neurotropism and neurovirulence are modulated by amino acid residues in and around the V3 loop subregion of the viral envelope, with the residue at the V3 loop position 5 correlating with neurocognitive deficit [34]. Several research teams have proposed CSF signatures or patterns that correlate with neurocognitive impairment within or in regions adjacent to the V3 loop [28,29,35,36]. Surface expression of CD4 on macrophages is considerably lower than on CD4+ T cells [37]. CNS-derived, macrophage-tropic HIV-1 isolates have demonstrated an abilty to infect cells expressing low levels of CD4 [38–40], attributed to alterations in gp120 engagement of the CD4 binding domain [41–43]. Dunfee et al. have previously identified an HIV *env* glycoprotein variant in the CD4-binding site of gp120 (N283) present at a high frequency in brain tissues from AIDS patients with HAD that enhances macrophage tropism and is associated with brain infection and dementia [44]. There is also evidence that CNS-derived HIV variants that efficiently infect macrophages may display greater affinity for the CCR5 HIV-1 co-receptor [45]. Though controversal, in the context of enhanced CD4-binding, the ability to utilize lower levels of CCR5 for macrophage entry is postulated to arise from a modified interaction between gp120 and CCR5 [46–48]. Since regions outside the V3 loop have been demonstrated to influence loss of infectivity, host range, and syncytium-forming ability of T-cell line-tropic HIV-1 recombinant virus [49], analysis of full-length HIV-1 *env* sequences has the potential to reveal novel residues that may contribute to HIV-1 neurotropism.

We hypothesized that full-length HIV-1 *env* sequence analysis would allow for the identification of viral characteristics that are distinctly representative of the virus’ localization to the CNS. We describe genetic features of HIV-1 *env* that correlate with the presence of viral variants in the CSF versus plasma, determine shared patterns of CNS compartmentalization of HIV-1 *env* in a cohort of individuals with chronic HIV-1 infection, reveal the existence of correlated mutation covariation across full length HIV-1 *env* and interpret these findings in the context of current knowledge of the structure of gp120. We generated viral sequences by single genome amplification (SGA), whereby PCR products are derived from a single template molecule, allowing for the most accurate representation of *in vivo* HIV-1 quasi-species for genetic/phylogenetic analysis [50,51]. Investigation of these parameters across individuals with normal neurocognitive performance (NCN) and importantly, those with either ANI or MND allowed for the focus on individuals with the most relevant HAND clinical diagnoses in the cART era.

## Results

### Clinical characteristics of the study groups

We obtained clinical samples from fifteen individuals with chronic HIV-1 infection previously enrolled in the CNS HIV Antiretroviral Therapy Effects Research (CHARTER) study. During enrollment in the study, these individuals underwent comprehensive neurocognitive testing and the Global Deficit Score (GDS) method was used to classify overall neuropsychological (NP) impairment status as previously described [52,53]. Validated cutpoints for NP impairment were used to classify individuals with NCN, ANI and MND. At the time of neurocognitive testing, contemporaneous cerebrospinal fluid (CSF) and peripheral-blood samples were obtained from each individual. De-identified, cryopreserved CSF and plasma samples were provided by CHARTER for this study. Participant demographic and clinical characteristics are detailed in Table 1.

**Table 1:** **Clinical and demographic profiles for study participants**

**Participant**	**NCN1**	**NCN2**	**NCN3**	**NCN4**	**NCN5**	**NCN6**	**ANI1**	**ANI2**	**ANI3**	**ANI4**	**ANI5**	**ANI6**	**ANI7**	**MND1**	**MND2**
**Age**	52	41	42	41	45	43	61	51	42	44	37	34	62	40	32
**Sex**	M	M	M	M	M	M	M	M	M	M	M	M	M	M	F
**Race/Ethnicity**	White	Black	Black	White	Black	White	White	White	Black	White	Hispanic	White	White	Black	Black
**Est. Dur. Inf**	59.6	173.1	48.9	214.4	75.3	136.9	154.1	211.7	46.2	56.2	26.5	59.8	206.6	75.0	58.5
**ARV Status**	No ARVs	Naïve	No ARVs	Naïve	Naïve	No ARVs	Naïve	No ARVs	Naïve	Naïve	Naïve	No ARVs	No ARVs	Naïve	No ARVs
**CD4 Count**	591	802	687	790	420	418	743	220	819	237	244	329	466	398	320
**CD4 Nadir**	400	554	491	461	297	392	537	220	495	237	244	329	215	398	320
**Plasma VL**	65,600	749	8,620	13,900	19,700	18,900	5,320	29,800	1,890	38,600	9,210	13,000	50,100	6,250	44,900
**CSF VL**	120	120	6,600	14,700	18,300	2,910	3,210	5,930	129	1320	653	2430	4,330	454	2,690
**RPR**	Neg	Neg	Neg	Neg	Neg	Neg	Neg	Neg	Neg	Neg	Neg	Neg	Neg	Neg	Neg
**HCV**	Neg	Neg	Neg	Neg	Neg	Neg	Neg	Neg	Neg	Neg	Neg	Neg	Neg	Neg	Neg

Participant neurocognitive disease classifications are as follows: NCN = Normal neurocognitive performance; ANI = Asymptomatic neurocognitive impairment; MND = Mild neurocognitive disorder. M = male, F = female. Estimated Duration of Infection (Est. Dur. Inf) is shown in months. CD4+ T Cell Count (CD4 Count) is in cells/mm^3^, plasma and CSF HIV-1 RNA levels (VL) are in copies/mL. RPR = rapid plasma reagin. HCV = hepatitis C virus.

Given that the ANI and MND diagnoses share their objective criteria, these two groups were combined for the purposes of statistical analysis of key demographic characteristics, and experimental comparisons to individuals with NCN. All neurocognitive groups were comprised of viremic subjects naïve to antiretroviral therapy, as well as those with a prior history of antiretroviral use who were not using cART at the time of the study visit (Tables 1 and 2). The majority of samples were from white, male participants. The mean age of individuals included in this study did not significantly differ amongst the groups. Groups also did not statistically differ in their estimated duration of infection, CD4+ T cell counts, plasma or CSF HIV-1 RNA levels (*p* > 0.05 for all comparisons) (Table 2). A CD4+ T cell nadir below 200 cells/mm^3^ has been statistically correlated with the onset of neurocognitive impairment [6]. Although the mean CD4+ T cell nadir was somewhat lower amongst the ANI and MND group than in individuals without neurocognitive impairment (333 versus 433 cells/ mm^3^) these differences were not statistically significant, and no individual in this study had a CD4+ T cell nadir below 200 cells/mm^3^ (Tables 1 and 2). In an effort to exclude potential confounders that may complicate the interpretation of HAND, cases were not included if it was clinically probable that neurocognitive impairment or disease was most likely attributed to co-morbidities other than HIV infection. As seen in Table 1, samples from individuals without evidence of chronic infection with hepatitis C as determined by a negative hepatitis C antibody test at the time of the neurocognitive testing and sample acquisition were chosen for analysis [54]. Additionally, none of the individuals studied had an HIV infection risk factor of injection drug use (IDU) or evidence of active syphilis (rapid plasma reagin (RPR) positive) at the time of sample collection.

**Table 2:** **Statistical comparison of key clinical and demographic parameters across study groups**

**Groups**	**NCN**	**ANI + MND**	***p-*** **value**
# Male Sex/total	6/6	8/9	N/A
# Men who have sex with men/total	5/6	7/9	N/A
Mean Age (years, range)	44 (41-52)	45 (32-62)	0.72
Mean Years of Education (years, range)	13 (10-16)	14 (9-18)	0.31
Mean Est. duration of infection (months, range)	118 (49-214)	99 (27-212)	0.46
Mean CD4+ T cell count (cells/mm^3^, range)	618 (418-802)	420 (220-819)	0.09
Mean CD4+ T cell nadir (cells/mm^3^, range)	433 (297-554)	333 (215-537)	0.15
Mean plasma HIV-1 RNA (log copies/mL, range)	4.3 (2.9-4.8)	4.3 (3.3-4.7)	1.00
Mean CSF HIV-1 RNA (log copies/mL, range)	3.9 (2.1-4.3)	3.4 (2.1-3.8)	0.52

All *p-*values determined by Mann Whitney test. For all comparisons, *p*-values <0.05 are considered significant.

### Single genome amplification of full length HIV-1 envelope

**Figure 1:**
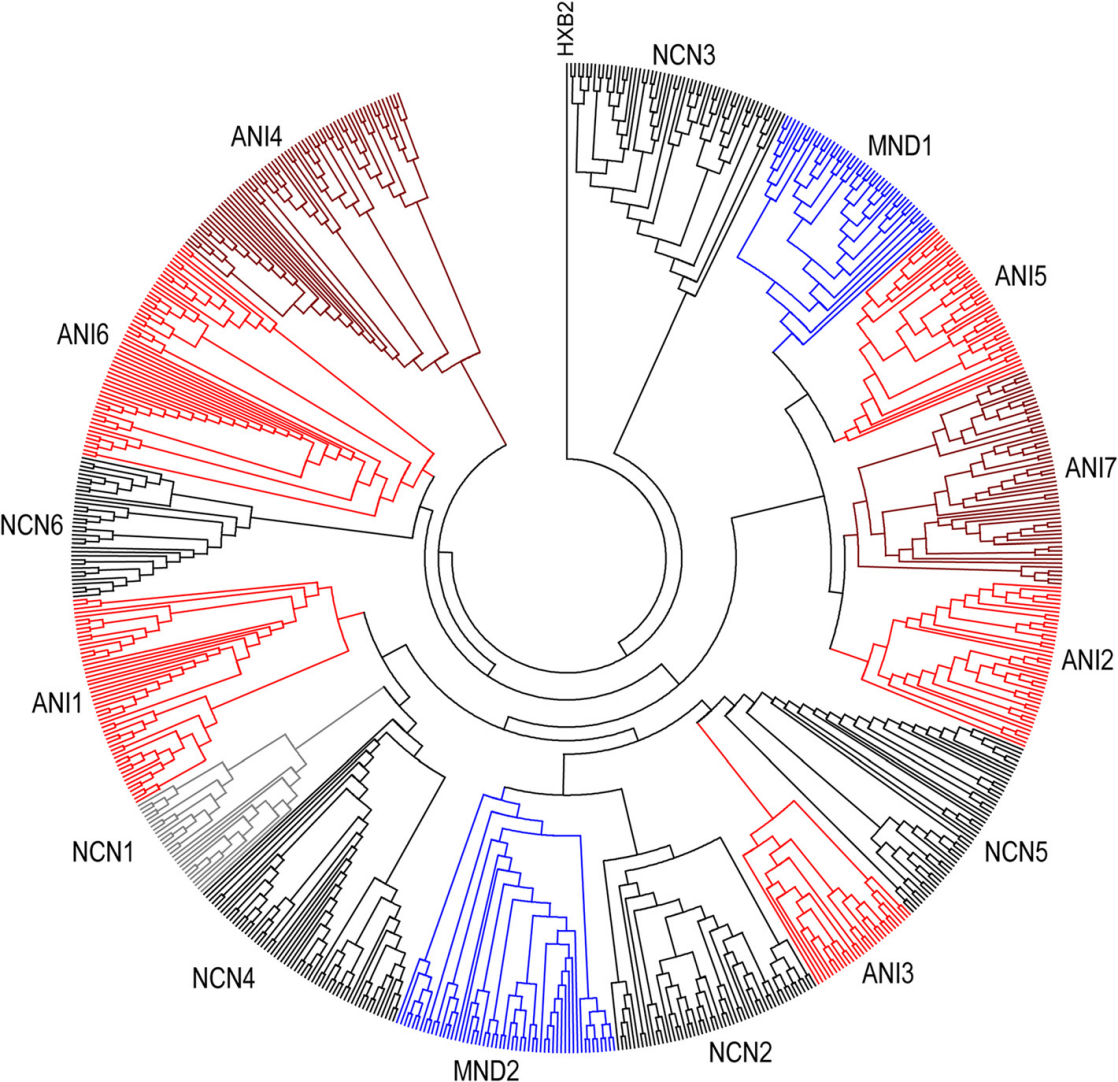
**Intra-Patient Clustering of HIV-1** ***env*** **quasi-species.** Maximum Likelihood (ML) topology view tree depicting full-length HIV-1 *env* sequences from fifteen experimental subjects is shown. For each subject, all sequences from both compartments (plasma and CSF) are shown. Each subject forms a tight cluster and is distinct from other experimental subjects with aLRT SH-like supports >95% for all inter-subject clusters. HXB2 was used as an outgroup.

Single genome amplification of HIV-1 *env* (>2.5 kb) was performed on viral RNA from contemporaneous, cryopreserved plasma and CSF samples for each individual using the method of Salazar-Gonzalez et al. [55]. All study participants were chronically infected with HIV-1 Subtype B virus as determined by the REGA HIV-1 subtyping tool [56,57]. A total of 717 confirmed single genome sequences (SGS) from fifteen (15) patients were obtained as described in Methods. Consistent with the absence of contamination between patient samples during PCR [58] phylogenetic analysis demonstrates that sequences from each patient form tight and distinct clusters (Figure 1).

### Significant HIV-1 *env* compartmentalization between the CSF and plasma is seen in a subset of individuals across neurocognitive disease states and cART status

**Figure 2:**
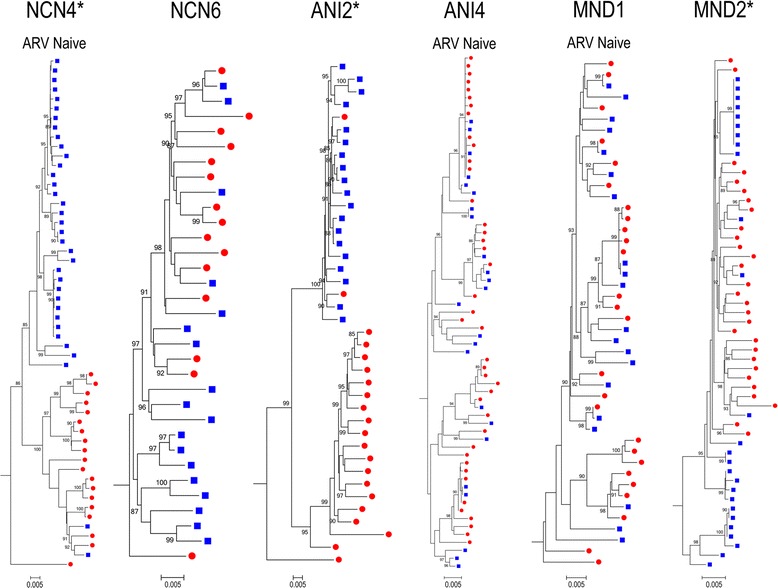
**HIV-1** ***env*** **phylogenies - Varying degrees of intra-patient sequence diversity and compartmentalized virus across disease states.** Representative ML trees of SGA sequences from select participants. For all panels, CSF (closed blue squares) and plasma (closed red circles) are shown. aLRT SH-like supports were determined and values over 85% are shown. HIV-1 *env* sequences with statistical evidence of hypermutation were excluded. All scale bars represent 0.005 nucleotide substitutions per site. HXB2 was used as an outgroup. * = Phylogram with statistical evidence of viral compartmentalization.

Phylogenetic analyses of HIV-1 *env* sequences have documented distinct viral populations in the CSF and blood compartments in both primary and chronic infection [20,34,59]. We therefore sought to identify individuals in whom genetic characteristics of HIV-1 *env* quasispecies in each compartment would allow us to distinguish CSF from plasma variants by performing formal analyses of compartmentalization. Nucleotide sequences were analyzed to increase the sensitivity of identifying genetic compartmentalization when present and two complementary methods were used. The multiple-alignment based, nonparametric test for panmixia [60] was derived from a geographic subdivision detection test proposed by Hudson et al. [61]. The phylogenetic-tree based Slatkin-Maddison (SM) test was used as a second, confirmatory test to infer true shifts in population structure in a sample [62] as implemented in HyPhy [63]. To avoid bias in the determination of compartmentalization, duplicate sequences within each compartment and sequences with statistical evidence of G-A hypermutation were removed from each patient’s dataset prior to analysis. In this cohort, 3 of 6 (50%) individuals with NCN and 5 of 9 (56%) with ANI or MND demonstrate statistical evidence of compartmentalization. When examined based on antiretroviral therapy treatment history, it is also apparent that compartmentalization of virus in the CSF can been seen in patients that are both naïve to cART (3/8) as well as those with prior treatment histories (5/8). Representative individual phylograms are shown in Figure 2 and the results of formal compartmentalization analysis for all studied subjects are shown in Table 3.

**Table 3:** **HIV-1** ***env*** **compartmentalization between the CSF and plasma is seen in a subset of individuals across disease states**

**Participant**	**Panmixia**	**Slatkin-Maddison**	**#CSF Sequences**	**#Plasma Sequences**
NCN1	0.5820	0.4585	7	20
NCN2	0.0486	0.0672	29	23
NCN3*	<0.0001	0.0002	23	27
NCN4*	<0.0001	<0.0001	35	19
NCN5*	0.0027	0.0066	27	19
NCN6	0.0011	0.1336	18	15
ANI1*	<0.0001	<0.0001	28	25
ANI2*	<0.0001	<0.0001	19	21
ANI3	0.3260	0.4889	12	17
ANI4	0.3840	0.1073	24	41
ANI5	0.0032	0.0821	22	19
ANI6*	<0.0001	<0.0001	30	28
ANI7*	<0.0001	<0.0001	31	23
MND1	0.2010	0.5451	20	26
MND2*	<0.0001	<0.0001	26	29

Panmixia and Slatkin-Maddison (SM) probabilities are shown for each study patient. #CSF and #Plasma Sequences = number of sequences used in analysis after exclusion of hypermutated sequences. For probability of panmixia and Slatkin-Maddison test, *p*-values <0.05 are considered significant. Starred patients (*) are those with statistically compartmentalized virus using both methods.

### CCR5-tropic virus predominates in the CSF and plasma in the majority of HIV-1+ individuals with varying degrees of neurocognitive impairment

The majority of CNS-derived HIV-1 strains use the chemokine CCR5 (R5) as the coreceptor for entry into macrophages and microglia [45,64]. HIV-1 strains able to use both CCR5 and CXCR4 for cellular entry (so-called dual-tropic or R5X4 strains) have less frequently been identified in the brains of some individuals [38,65].

We therefore hypothesized that the majority of SGA-derived HIV-1 *env* variants in the CSF and plasma of individuals with NCN, ANI and MND would exhibit CCR5 tropism, anticipating a minority of variants predicted to use CXCR4. Translated V3 loop sequences were scored using Geno2Pheno [66] and the SINSI position-specific scoring matrix [PSSM] prediction algorithm [67]. A Geno2Pheno false-positive rate (FPR) (1-specificity) of 5% was chosen based on reports using similar FPRs to derive co-receptor predictions that when compared with results from the Monogram Trofile assay, resulted in similar clinical response rates to the CCR5 inhibitor maraviroc [68]. PSSM has a reported sensitivity of 84% and 96% specificity for the prediction of CXCR4 usage [67].

CCR5-tropic virus was found to predominate in the CSF and plasma in the majority of HIV-1+ individuals studied, irrespective of neurocognitive disease classification (Table 4). In general, co-receptor tropism classifications were in agreement using the two methods. Concordant results using both methods identified rare instances of variants with a predicted ability to use CXCR4 in the plasma of two individuals with ANI (ANI1 and ANI2). Discordant results for participant ANI7 suggest infrequent (PSSM) or no (Geno2Pheno) CXCR4-using variants in the CSF and plasma. Interestingly, we were never able to identify CXCR4-using variants in the CSF in the absence of similarly classified plasma variants although the reverse was true. Patient ANI3 was found to have a predominance of variants with predicted ability to use CXCR4 in both the CSF and plasma. Genotypic changes allowing the virus to use CXCR4 have been associated with the more rapid progression of HIV-1 disease [69]. However, the extent to which the predominance of CXCR4-using virus in this individual with a well-preserved CD4+ T cell count, relatively low CSF and plasma HIV-1 viral load (Table 1) and no evidence of compartmentalized virus may have influenced their neurocognitive status is unclear.

**Table 4:** **CCR5-tropic virus predominates in the CSF and plasma in the majority of HIV-1+ individuals with varying degrees of neurocognitive impairment**

**Participant**	**Compartment**	**G2P CCR5**	**G2P CXCR4**	**PSSM CCR5**	**PSSM CXCR4**	**No Prediction**
NCN1	CSF	7	0	7	0	0
	Plasma	20	0	20	0	0
NCN2	CSF	29	0	29	0	0
	Plasma	23	0	23	0	0
NCN3	CSF	26	0	26	0	0
	Plasma	29	0	29	0	0
NCN4	CSF	35	0	35	0	0
	Plasma	22	0	22	0	0
NCN5	CSF	28	0	28	0	0
	Plasma	21	0	21	0	0
NCN6	CSF	18	0	18	0	0
	Plasma	17	0	17	0	1 G2P + PSSM (Ins)
ANI1*	CSF	28	0	28	0	0
	Plasma	23	2	23	2	0
ANI2*	CSF	20	0	20	0	0
	Plasma	20	1	20	1	0
ANI3*	CSF	0	12	0	12	0
	Plasma	1	16	1	16	0
ANI4	CSF	24	0	24	0	0
	Plasma	40	0	41	0	1 G2P (Del)
ANI5	CSF	22	0	22	0	0
	Plasma	19	0	19	0	0
ANI6	CSF	30	0	30	0	0
	Plasma	27	0	28	0	1 G2P (Del)
ANI7*	CSF	31	0	29	2	0
	Plasma	23	0	22	1	0
MND1	CSF	20	0	20	0	0
	Plasma	26	0	26	0	0
MND2	CSF	26	0	26	0	0
	Plasma	29	0	29	0	0

The number of translated V3 loop sequences predicted to be CCR5 and CXCR4-tropic using both the Geno2Pheno (G2P) and the SINSI position-specific scoring matrix (PSSM) for all sequenced viral variants are shown. Three sequences yielded invalid predictions on co-receptor usage as a result of insertions (Ins) or deletions (Del) in the translated V3 loop sequence. Individuals for whom CXCR4-tropic variants are predicted in the CSF or plasma compartments are starred (*).

### Amino acid diversity is lower across compartmentalized CSF-derived full-length HIV-1 *env*

**Figure 3:**
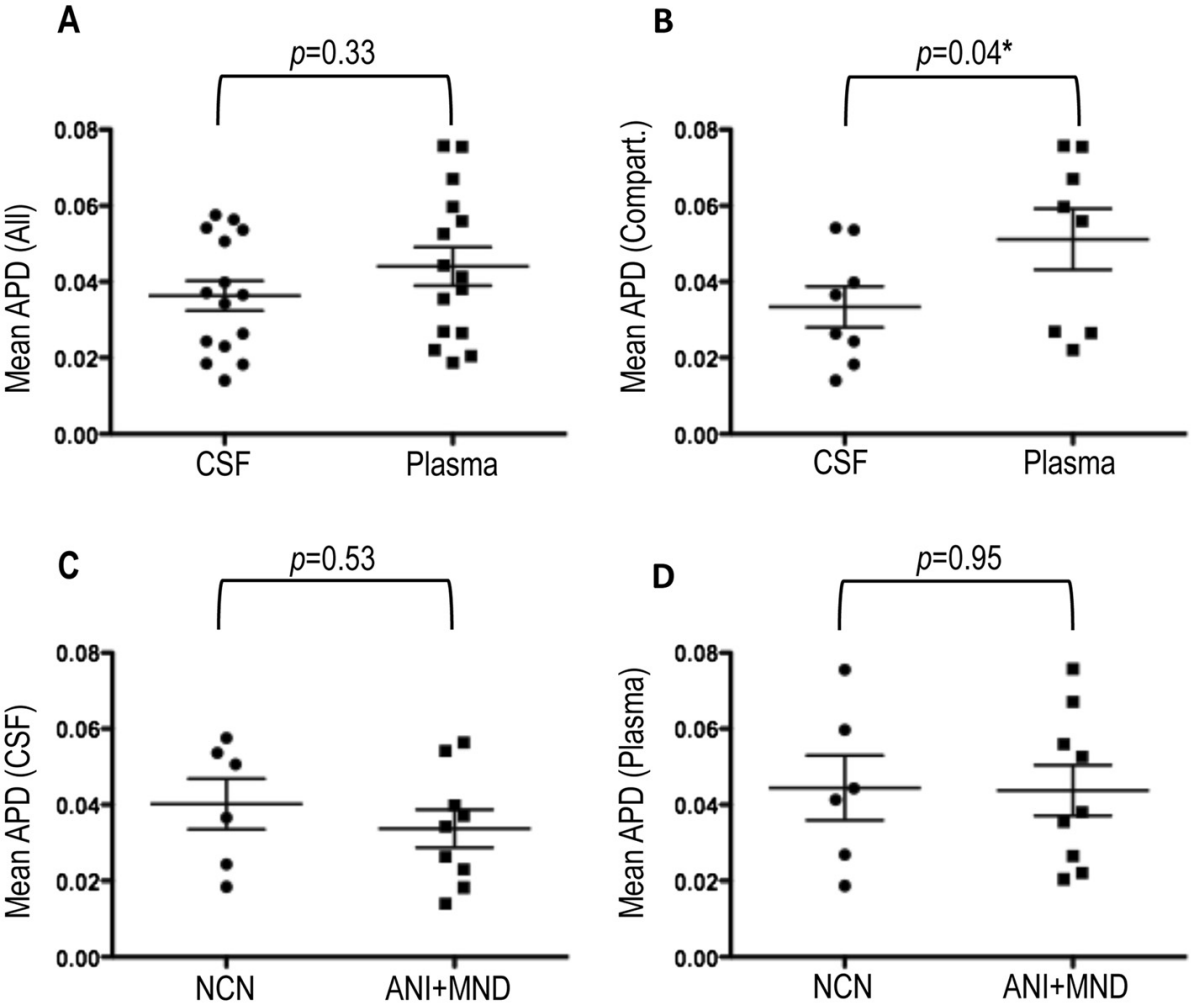
**Mean population amino acid diversities across HIV-1** ***env.*** Mean Average Pairwise Distance determinations between the **(A)** paired CSF and plasma HIV-1 *env* variants of all participants (n = 15) and **(B)** CSF and plasma HIV-1 *env* variants of participants with statistical evidence of CSF viral compartmentalization (n = 8) **(C)** CSF of individuals with NCN (n = 6) and those with neurocognitive disease (ANI + MND) (n = 9) and **(D)** plasma of individuals with NCN (n = 6) and those with ANI or MND (n = 9). All calculations were performed after the exclusion of sequences with statistical evidence of hypermutation. The Wilcoxon matched-pairs signed rank test and the Mann Whitney test were used to determine *p-*values for paired observations (CSF vs. plasma) and observations between disease classifications (NCN vs ANI + MND) respectively. APD = Average Pairwise Distance. For all comparisons, *p*-values <0.05 are considered significant. * = Statistically significant

The blood-brain barrier (BBB) plays a critical role in preserving immune privilege in the CNS [70] and prior studies using partial HIV-1 *env* have found lower viral sequence diversity in the CSF compared to plasma [34,71]. We therefore hypothesized that full-length HIV-1 *env* amino acid diversity would be lower in CSF-derived quasispecies when compared to their plasma counterparts. The generation of multiple single genome HIV-1 *env* variants from each compartment allowed us to look specifically at the amino acid population diversity present in paired plasma and CSF compartments. Using multiple alignments of full-length HIV-1 *env* patient sequences after the exclusion of variants with statistical evidence of hypermutation, mean average pairwise distances (APD) were determined for each quasispecies of interest, providing a measure of population diversity. Mean HIV-1 *env* population diversities in both the CSF and plasma compartments were consistent with chronic HIV infection. Irrespective of neurocognitive disease classification, in the group of individuals with evidence of compartmentalized viral variants in the CSF (n = 8), in paired observations, mean diversity of CSF quasispecies was significantly lower than that seen in plasma collected at the same time point (3.34% vs. 5.12%, *p* = 0.04) (Figure 3B). In the absence of compartmentalization, these differences are not statistically significant (3.63% vs. 4.40%, *p* = 0.33) although this study is not powered to detect small magnitude differences (Figure 3A). We were similarly interested in investigating differences between the level of quasispecies diversity in the CSF and plasma across disease states. For these analyses, we compared individuals with NCN (n = 6) to individuals in the neurocognitively impaired (ANI and MND) groups combined (n = 9). Mean HIV-1 *env* population diversities were not statistically different between individuals with NCN and those with ANI + MND in either compartment (4.02% vs. 3.37%, *p =* 0.53 in CSF and 4.44% vs. 4.38%, *p =* 0.95 in plasma) (Figure 3C-3D).

While not true for all individuals, it is often observed that measured HIV-1 RNA levels in the CSF are anywhere from 1-2 log_10_ lower than those measured in contemporaneous plasma [72,73]. In an attempt to determine if the differences observed in genetic diversity (APD) of the paired CSF and plasma compartments in the group was simply a refection of differences in HIV-1 RNA levels (VL) within the compartments, linear regression analysis comparing the ratio of each individuals’ plasma to CSF APD to the log of the ratio of the plasma to CSF VL was performed. Linear regression analysis revealed the absence of any significant correlation between the two ratios (r^2^ = 0.10, *p* = 0.24), suggesting that factors other than HIV-1 VL are responsible for the observed genetic divergence between paired CSF and plasma compartments in this study (Additional file 1: Figure S1).

### Variation in the degree of genetic divergence between CSF and plasma variants across full length HIV-1 *env*

**Figure 4:**
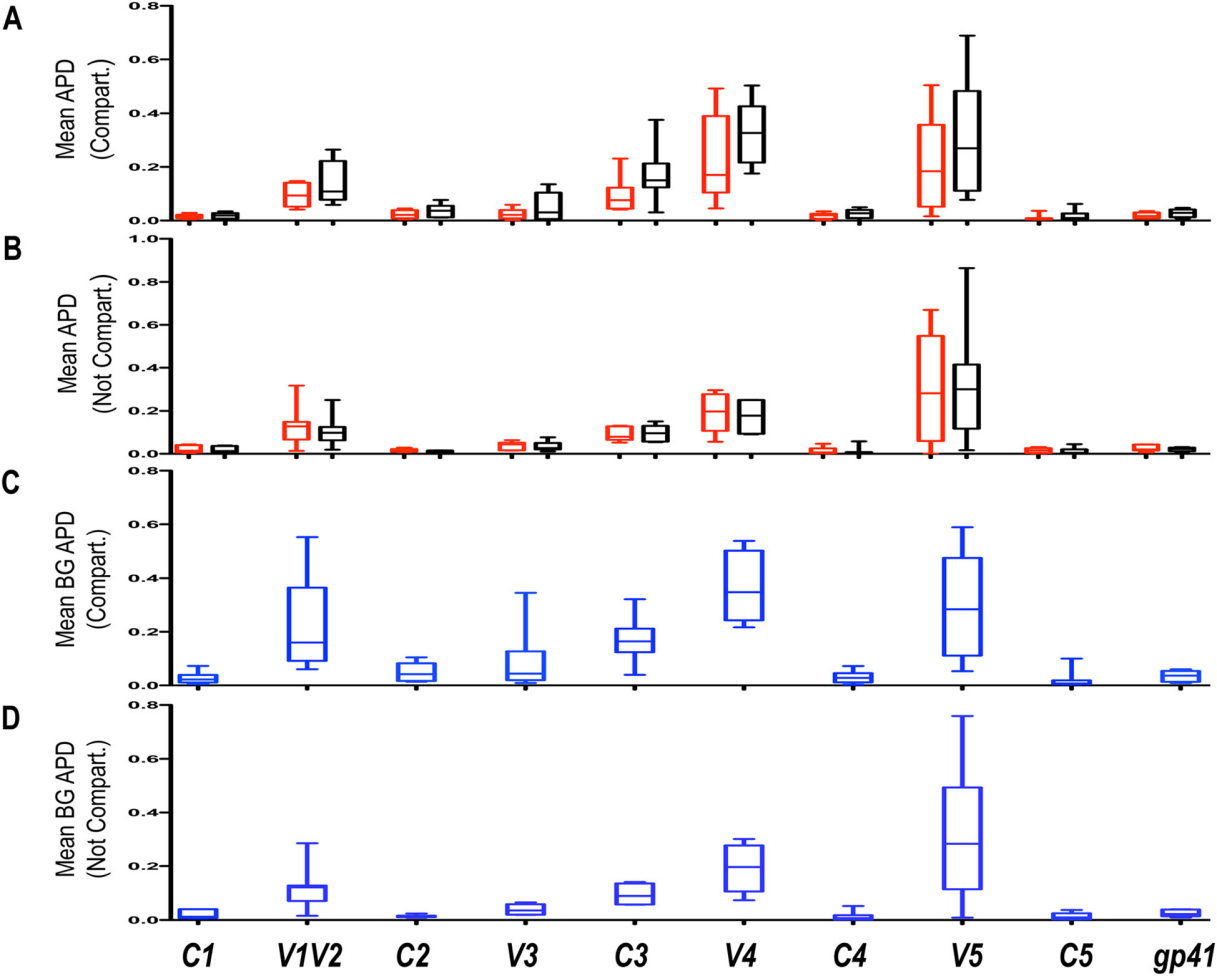
**Variation in amino acid genetic diversity across HIV-1** ***env*** **.** Box plots displaying Mean Average Pairwise Distances (APD) between the **(A)** paired CSF (red) and plasma (black) variants of subjects with statistical evidence of CSF viral compartmentalization (Compart.) across all regions of HIV-1 *env* (n = 8) and **(B)** paired CSF (red) and plasma (black) of variants of subjects without statistical evidence of CSF viral compartmentalization (n = 7). In panel **(C)** the Mean Between Group (CSF vs. Plasma) APD is shown for subjects with statistical evidence of CSF viral compartmentalization across all regions of HIV-1 *env* (n = 8) and in **(D)** the Mean Between Group (CSF vs. Plasma) APD is shown for subjects without statistical evidence of CSF viral compartmentalization across all regions of HIV-1 *env* (n = 7). All calculations were performed after the exclusion of sequences with statistical evidence of hypermutation. The Wilcoxon matched-pairs signed rank test was used to determine *p-*values for paired observations. APD = Average Pairwise Distance. For all comparisons, *p*-values <0.05 are considered significant. * = Statistically significant.

HIV-1 *env* consists of variable and constant regions, so termed because of the level of genetic variation within the region [74]. In Figure 4A, average genetic diversity within CSF and plasma populations as measured by APD in amino acid alignments are shown. Irrespective of disease classification, in individuals with evidence of viral compartmentalization (n = 8), a consistent trend towards lower genetic diversity in the CSF compared to paired plasma variants was seen in the variable regions of HIV-1 *env* as well as in the C3 region (Figure 4A). These differences were not, however, statistically significant when applying a stringent correction for multiple testing (Bonferroni adjusted *p-*value threshold of *p* = 0.005). As expected, in individuals without evidence of viral compartmentalization (n = 7), the trend towards decreased CSF genetic diversity was largely absent, reflecting the more equilibrated viral populations in these individuals (Figure 4B). In Figure 4C, mean APDs between CSF and plasma populations in individuals with evidence of viral compartmentalization are shown. While mean differences in genetic diversity between the CSF and plasma compartments (groups) are generally highest in the variable regions of HIV *env*, measurable differences in APD between compartments are also seen in the C3 region. In Figure 4D, the mean APD between the CSF and plasma compartments of individuals without evidence of compartmentalized virus again demonstrate a blunting of the between group distances, consistent with equilibrated virus. However, even in individuals without evidence of compartmentalized virus, the mean APD between compartments in the V1V2, V3, C3, V4 and V5 regions suggests that while generally equilibrated, across HIV-1 *env*, some CSF variants in these individuals contain residues that allow for their discrimination from their plasma counterparts.

### Statistically significant differences in the V1V2 loop length are seen in the CSF and plasma variants and correlate with differences in the number of potential N-linked glycosylation sites across the region

*In vivo*, the HIV *env* glycoproteins are the primary targets for neutralizing antibodies [75]. The surface proteins of HIV-1 are highly variable and highly glycosylated [76] and N-linked glycosylation on the HIV-1 *env* glycoprotein is a major mechanism for minimizing the virus neutralizing antibody response [77].

**Figure 5:**
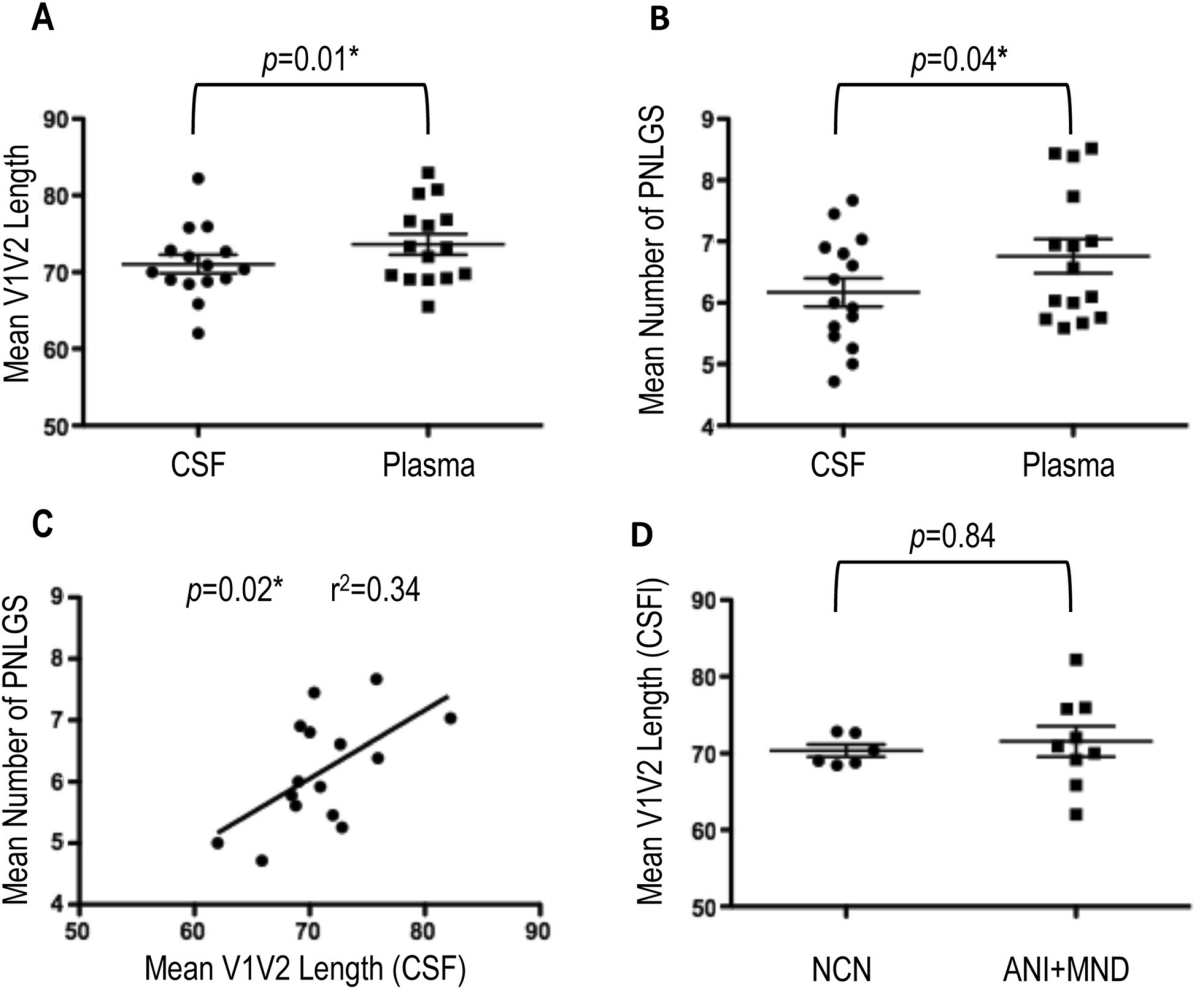
**V1V2 loop length and N-linked glycosylation (CSF vs Plasma).** The mean **(A)** amino acid lengths and **(B)** number of potential N-linked glyosylation sites (PNLGS) of the HIV-1 *env* V1V2 region from paired CSF and plasma quasispecies are shown for all subjects (n = 15). **(C)** Linear regression of the mean amino acid lengths of the HIV-1 *env* V1V2 region from CSF quasispecies with the corresponding mean number of PNLGS in the same quasispecies. **(D)** The mean amino acid lengths of the HIV-1 *env* V1V2 region in the CSF quasispecies of individuals with NCN and those with ANI + MND. The Wilcoxon matched-pairs signed rank test and the Mann Whitney test were used to determine *p-*values for paired observations (CSF vs. plasma) and observations between disease classifications (NCN vs ANI + MND) respectively. The linear regression score (r^2^) was derived in PRISM. For all comparisons, *p*-values <0.05 are considered significant. * = Statistically significant.

We therefore hypothesized that on average, CSF-derived variants would exhibit shorter V1V2 lengths and lower degrees of N-linked glycosylation than their paired plasma counterparts. To determine differences in V1V2 lengths between the CSF and plasma compartments, translated amino acid alignments were generated for each individual (n = 15). With the exception of those with evidence of G-A hypermutation, all patient-derived SGS were included in the analysis. Once aligned, the V1V2 region (amino acids corresponding to positions 131-196 relative to HIV-1 gp160 start in HXB2) was extracted for analysis. The mean amino acid length for all V1V2 variants was determined for each patient in the CSF and corresponding plasma compartment using MEGA [78]. As shown in Figure 5A, V1V2 loop lengths were significantly shorter in CSF than paired plasma variants (71.1 vs. 73.6, *p* = 0.01) across neurocognitive disease states. No difference was found in the V1V2 loop length of CSF variants from individuals with NCN when compared to individuals with ANI or MND (70.3 vs. 71.6, *p =* 0.84) (Figure 5D). The N-Glycosite program [79] was then used to identify PNLGS across HIV-1 *env* V1V2. In paired comparisons between CSF and plasma derived variants, the mean number of PNLGS was statistically lower in the CSF (6.2 vs. 6.8, *p* = 0.04) (Figure 5B). Linear regression analysis revealed a significant positive correlation between the V1V2 length in CSF variants and the mean number of PNLGS sites therein (*p* = 0.02) (Figure 5C). The linear regression score (r^2^ = 0.34) suggests a mild relationship, with approximately 34% of the difference in mean PNLGS being explained by the variation in V1V2 loop length.

### Molecular patterns of compartmentalized HIV-1 *env* reveal genetic signatures of CNS adaptation

We hypothesized that full-length HIV-1 *env* sequence analysis would identify sites in amino acid alignments that are distinctly representative of the virus’ localization to the CNS and that the use of SGA-derived full-length HIV-1 *env* would allow for the greatest accuracy and breadth of these analyses. The goal of these analyses was to identify positions in each individual along HIV-1 *env* that were selected with a high degree of statistical significance for compartmentalization. This would then allow us to determine if particular amino acid residues were favored for compartmentalization across individuals. Single genome sequences from all 15 individuals in the study were grouped into one master alignment containing the HXB2 HIV-1 *env* reference sequence, which was then translated in frame, resulting in an amino acid alignment. As was done for determinations of compartmentalization, duplicate sequences within individual patient compartments were removed from the analysis in an attempt to limit bias resulting from the analysis of clonally derived sequences. Sequences with evidence of statistically significant G-A hypermutation were also excluded. All non-HXB2 sites were removed from the alignments. For each individual, signature pattern analysis was performed using the Viral Epidemiology Signature Analysis (VESPA) software [80]. The VESPA software examines amino acid differences between groups of sequences (CSF SGS and plasma SGS). Positions where the dominant amino acid in the CSF alignment (query) was different than the dominant amino acid in the plasma alignment (background) were identified. A fisher’s exact test was then performed for each site to determine locations across HIV-1 *env* where statistically significant differences were noted in the CSF versus dominant plasma residue. A Bonferroni correction for multiple comparisons was performed for each participant’s alignment where the number of variable sites in their individual HIV *env* alignment was used to determine the correction. The number of individual SGS included in each alignment, variable sites per patient alignment calculated in MEGA [78], and the corresponding corrected Bonferroni *p-*value thresholds for significance are shown in tabular form as Additional file 2: Table S1. *P-*value thresholds ranged from *p* < 7×10^-4^ to *p* < 2×10^-4^.

In an attempt to exclude positions for which uncertainties in the multiple alignments might impact the results, we employed the GUIDANCE web-server [81]. Using bootstrap trees as guide-trees to the alignment algorithm, the GUIDANCE program constructs a set of multiple sequence alignments, measures the robustness of the alignment to guide-tree uncertainty and compares them to the base alignment in order to estimate its confidence level. In this way the tool identifies columns that are unreliably aligned, enabling their removal from the alignment. This comparison results in scores between 0-1 for each column of the multiple sequence alignment. Columns with a GUIDANCE score below 0.9 were excluded from further analysis. All reported amino acid positions are represented in a minimum of two independent participant alignments with a high degree of statistical significance.

**Figure 6:**
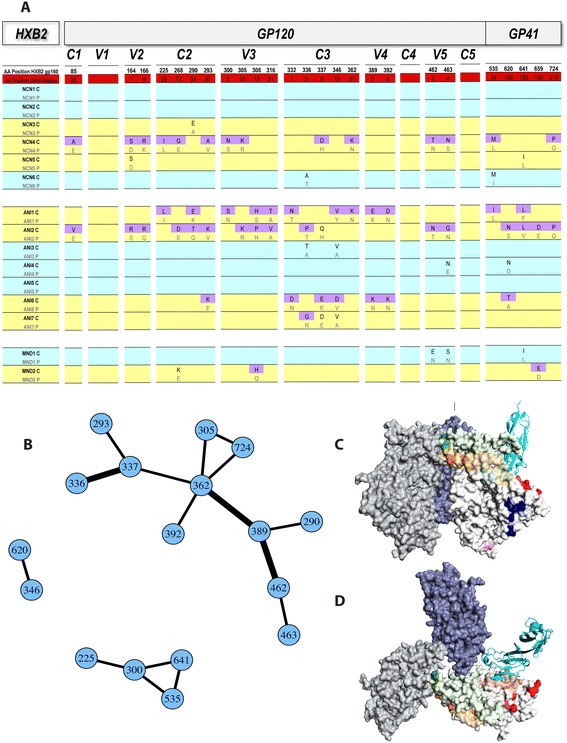
**Compartmentalization “Hot spots” across full-length HIV-1** ***env*** **and the network of correlated mutations.** Results of analyses identifying compartmentalization hot spots **(A)**. All positions listed are significant in ≥ 2 individuals. Subjects with statistically non-compartmentalized (blue) and compartmentalized (yellow) CSF quasispecies are shown. For each hot spot, corresponding HXB2 gp160 (white) and specific gene-region (C1-gp41) numbering (red) is shown (regions not drawn to scale). Amino acids at hot spot positions in CSF (C) alignments meeting the threshold for statistical significance are shaded in purple. Corresponding plasma (P) amino acids are shown directly below. **(B)** An adjacency matrix demonstrating hot spot positions with amino acids sharing statistically significant mutual information (MI) in ≥ 2 subjects. Lines connect positions sharing MI, with the thickness of the line corresponding to the number of studied subjects in which the correlation was identified. Surface representation of the hot spots on a gp120 trimer based on the SOSIP crystal structure in a side **(C)** and top **(D)** view. The three gp120s in the trimer were rendered as surfaces and colored light grey, grey and blue, respectively. For simplicity, the hot spot residues were colored individually and only displayed in one of the gp120s (light grey). A CD4 molecule (with N-terminal D1D2 domains; cyan ribbon) was placed onto this gp120 by superimposition of its complex with a gp120 core to indicate the location of the CD4 binding site (light red). The entire V1V2 region and V3 region are colored light green and light orange, respectively, while the hot spots are colored more intensely. Note that the hot spot residues form several spatial clusters on the gp120 surface, including the V2 tip region (dark green), V3 region (dark orange), the CD4 binding site proximal region (red), the outer domain cluster (blue) and the inner domain cluster (pink).

Figure 6A displays the compartmentalization hot spots identified along the full-length HIV *env*. As would be expected, while compartment discriminating positions can be seen in some individuals with non-compartmentalized (equilibrated) virus, none of these positions met statistical significance as defined above. The absence of discriminating positions in the V1 region is a reflection of the removal of uncertain columns in the master alignment. The majority of reported hot spot sites were shared by a maximum of two individuals in the study, representing 25% of individuals with compartmentalized virus (n = 8). Amino acid signature pattern analysis identified two CSF-specific residues in 3/8 compartmentalized individuals (37.5%), C2 position 97 (HXB2 gp160 position 293) and V3 position 13 (HXB2 gp160 position 308). Several researchers have reported the overrepresentation of particular amino acids at one or both of these positions in CSF-derived variants [28,30,34]. It is important to note that overall, amongst the 20 robust discriminating positions identified in the external glycoprotein gp120, we report several novel sites, particularly those outside of the C2-C3 region. An additional 5 novel positions are reported in the transmembrane glycoprotein gp41.

### A subset of amino acid residues that correlate with CSF compartmentalization are statistically correlated to one another

We further hypothesized that amino acids at a subset of these compartmentalization hot spots might mutate coordinately. Our derivation of full-length HIV-1 *env* CSF variants derived from single genomes allowed us to test this hypothesis. The R package program CorMut provides functions for detecting correlated mutations among specific amino acids [82]. This package was used to compute correlations among the amino acids in the CSF compartmentalization hot spot sites identified in the study cohort. Similar to the analyses of compartmentalization sites, correlation mutation analyses were performed independently for each individual in the study using the participants’ own codon-aligned plasma consensus sequence (derived from their plasma SGS variants) as a comparator to their multiple, codon-aligned CSF-derived SGS variants. For each individual, positions under consideration were restricted to those previously identified as being statistically significantly selected in CSF compartmentalization. This method allowed for the determination of CSF hot spot mutations sharing mutual information within each individual. In correlation analyses, the mutual information score (MI) expresses the measure of the strength of association between the two positions. An MI score of 0 suggests that the two positions are independent and that information in one position provides no information about the other. An MI score of 1 suggests that all information conveyed by one position is shared with the other. For these analyses, an MI score threshold of 0.10 was chosen. As an additional criteria, mutations were considered significantly correlated if the Benjamini–Hochberg adjusted *p*-value for the correlation was less than 0.05 (corresponding to a 5% false discovery rate). Finally, as with the compartmentalization analyses, all reported correlated mutations were identified in at least 2 independent observations (study individuals) in an attempt to increase the chance that reported associations were the result of immune pressure as opposed to resulting from founder effect. The network of correlated mutations, number of individuals sharing the correlation and complete listing of amino acids in each pair of sites exhibiting mutual information are displayed in Figure 6B, Table 5, and Additional file 3: Dataset S1. Correlation between C3 position 5 (HXB2 gp160 336) and C3 position 6 (HXB2 gp160 337), C3 position 31 (HXB2 gp160 362) and V4 position 5 (HXB2 gp160 389), and V4 position 5 (HXB2 gp160 389) and V5 position 3 (HXB2 gp160 462) were the most-often identified correlations, each noted in 3 of 15 (20%) individuals studied. While these short-range correlations are most expected, this analysis also reveals significant correlation between amino acid positions in V3 and gp41, as well as positions in C3 and gp41. The V3 position 5 (HXB2 gp160 300) was found to share a significant degree of MI with three other positions; C2 position 29 (HXB2 gp160 225) and gp41 positions 24 and 130 (HXB2 gp160 535 and 641). C3 position 31 (HXB2 gp160 362) was found to share significant MI with 5 other hot spot positions, the greatest number identified in this analysis. In contrast, a number of positions statistically linked to CSF compartmentalization in at least 2 individuals were not found to covary significantly with any of the other such identified positions in at least 2 individuals studied. These positions include those closest to the N-terminus of gp160 (C1 position 85, V2 positions 164 and 166).

**Table 5:** **The network of correlated mutations in compartmentalization hot spot positions across HIV-1** ***env***

**aa1**	**aa2**	**Patients**
225 (C2 29)	300 (V3 5)	NCN4, ANI1
290 (C2 94)	389 (V4 5)	NCN4, MND2
293 (C2 97)	337 (C3 6)	NCN4, ANI6
300 (V3 5)	535 (gp41 24)	NCN2, ANI1
300 (V3 5)	641 (gp41 130)	NCN2, ANI1
305 (V3 10)	362 (C3 31)	NCN4, NCN5
305 (V3 10)	724 (gp41 213)	NCN4, NCN5
336 (C3 5)	337 (C3 6)	NCN3, ANI2, MND1
337 (C3 6)	362 (C3 31)	NCN4, MND1
346 (C3 15)	620 (gp41 109)	ANI1, ANI7
362 (C3 31)	389 (V4 5)	ANI1, MND1, MND2
362 (C3 31)	392 (V4 8)	NCN5, ANI1
362 (C3 31)	724 (gp41 213)	NCN4, NCN5
389 (V4 5)	462 (V5 3)	ANI4, MND1, MND2
462 (V5 3)	463 (V5 4)	ANI2, MND1
535 (gp41 24)	641 (gp41 130)	NCN2, ANI1

Compartmentalization Hot spots with amino acids sharing mutual information (MI) in ≥ 2 subjects, an MI value of ≥ 0.10 and a Benjamini–Hochberg adjusted *p*-value for the correlation < 0.05. HXB2 gp160 numbering for amino acids (aa1 and aa2) followed by corresponding gene-region specific amino acid positions are shown.

### 3D spatial relationships of the compartmentalization hot spot positions in the HIV-1 *env* trimer

To gain a functional understanding of the compartmentalization hot spots, we projected these positions onto the recently published crystal structure of the SOSIP trimer, which is a stabilized gp140 crystallized in complex with the Fab of PGT122 [83]. Due to the limited resolution of the structure, only residues in gp120 were identified (Figures 6C and 6D). The hot spot residues can be naturally grouped into several spatial clusters: (1) The V2 tip cluster (residues 164 and 166); (2) the V3 cluster (residues 300, 305, 308, and 316); (3) the cluster proximal to the CD4 binding site (residues 362, 389, 392, 462, and 463); (4) the inner domain (residue 85 and 225); (5) and outer domain (residues 268, 290, 293, 332, 336, 337, and 346) clusters. These 5 clusters are likely also functionally distinct. The residues in the V2 tip cluster are located at the tip of the strands B and C of V1V2 [84] which is packed against the other 2 molecules in the trimer (trimer association), thus they will likely play a role in the trimer formation. The residues in the V3 cluster are packed against V1V2, and will influence the interaction with V1V2. They may also be involved in co-receptor binding. Although the cluster proximal to the CD4 binding site does not overlap with the CD4 binding site, it is next to it and will likely influence CD4 binding site formation. The inner domain cluster has only two residues; one (residue 85) located on the inner domain surface and the other (residue 225) buried in the core. Residues in this domain have been shown to influence the layer formation of gp120 [85] thus the inner domain cluster can play similar roles. The outer domain cluster is the largest cluster of the hot spot residues and is located on the other side of the CD4 binding site. As the majority of CD4 binding residues are in the outer domain of gp120, it is conceivable that changes in residues in this cluster situated on the back of the outer domain may influence the formation of the CD4 binding site.

## Discussion

Combination antiretroviral therapy (cART) has become the standard of care for the treatment of HIV-1 infection, and can effectively and persistently suppress viral replication, as reflected by the reduction of plasma HIV-1 RNA to levels below detection in adherent patients. As such, progression of HIV infection to AIDS and death have been dramatically reduced with a considerable decrease in morbidity [86,87]. Similarly, the widespread use of cART has also led to a clear reduction in the incidence of HIV-associated dementia (HAD), one of the most severe manifestations of HIV-1 CNS infection. Despite this decrease, the prevalence of less severe HIV-1 associated cognitive impairment appears to be on the rise [8,88,89].

HIV-1 exhibits significant genetic diversity that is not equally distributed across the genome [90]. The most dramatic features of variability are localized to the viral *env* gene, particularly the five variable regions of gp120 (V1-V5) [91]. In this study we have performed a detailed comparative genetic examination of the distinct mutational patterns exhibited by HIV-1 *env* variants in the plasma and CSF of fifteen individuals chronically infected with HIV-1 Subtype B. In the analyses, cerebrospinal fluid (CSF) virus was used as an investigative surrogate for brain-derived HIV-1. Although indirect, this strategy is validated by phylogenetic evidence that CSF and brain-derived viral populations are more closely related to each other than to populations derived from other body compartments [92]. We hypothesized that detailed analyses of full-length HIV-1 *env* (>2.5Kb) would allow for the identification of genetic characteristics associated with the presence of viral variants in the CNS. We analyzed 717 confirmed single genome sequences (SGS) and demonstrate that measurable variation exists in the degree of genetic divergence between CSF and plasma variants across full length HIV-1 *env***,** various degrees of compartmentalization between the CSF and plasma variants exist across neurocognitive disease states and in individuals with compartmentalized virus, mean HIV-1 *env* diversity is significantly lower in CSF- than in plasma-derived variants.

The antibody response to HIV infection evolves in concert with viral diversity, resulting in the emergence of neutralization-resistant HIV variants [77]. Several studies have found that efficient replication of HIV-1 in macrophages and microglia correlates positively with increased sensitivity to neutralizing antibodies [41,45,93] and neurotropic HIV-1 isolates with increased CCR5 affinity have been demonstrated to be more sensitive to antibody neutralization [41,45]. In comparison to plasma-derived virus, the extent of N-linked glycosylation across the C2-V3 *env* subregion has been shown to trend somewhat lower in viral variants cloned from the CSF [34]. More recently, the neutralization resistance of a reference panel of tier-categorized neutralization-sensitive and resistant HIV-1 plasma-derived variants has been demonstrated to correlate with a longer V1V2 loop containing more potential N-linked glycosylation sites (PNLGS) [94]. When considered in concert with our findings of decreased mean diversity in CSF-derived full-length HIV-1 *env* quasispecies - a finding consistent with prior studies using partial HIV-1 *env* [34,71] - our findings that the mean V1V2 loop length is shorter and the mean number of PNLGS is lower in CSF-derived variants compared to their paired plasma counterparts supports the hypothesis that immune selection pressures are reduced in this privileged compartment [70]. The significant positive correlation between V1V2 loop length and number of PNLGS in CSF variants is evidence that length variation in the V1V2 loop is a tool for evolutionary selection. The correlation r^2^ of 0.35 between the two parameters, however, suggests that the degree of glycosylation seen in this region is not solely the direct reflection of V1V2 loop length and that other immune factors, such as potential selection pressures related to macrophage tropism, may play an important role. The previous finding by Drunfee et al. that targeted loss of N-linked glycosylation at position 386 in the V4 region enhances macrophage tropism and is associated with dementia support this possibility [95].

Published studies specifically seeking to reveal specific sites of HIV-1 *env* that distinguish them from plasma counterparts have typically been performed using either the heteroduplex tracking assay (HTA) [31] or bulk and near-endpoint PCR followed by cloning and sequencing [34]. The use of SGA allows for a more accurate representation of *in vivo* CSF specific HIV-1 quasi-species. Using this method, we took a unique approach to the identification of HIV-1 *env* positions in intra-patient phylogenies where the dominant amino acid differs significantly between CSF and plasma quasispecies in both variable and constant regions of gp120 as well as in gp41. Comparing positions across individuals, we identified 25 compartmentalization hot spots across the full-length envelope gene. These include multiple novel positions in HXB2 gp160, including those at 463 (V5 4) and 535 (gp41 24) as well as at the previously identified position 308 (V3 13), which has been reported in several studies identifying compartmentalization sites using consensus population sequences [28,30,34]. In addition, the presence of certain residues at position 308 has been associated with macrophage tropism [96] and dementia in studies of autopsy brain samples from those with HIV [29]. The presence of a hot spot at position 362 (C3 31) is noteworthy, as this potential N-linked glycosylation site (N362) seen here in the plasma of two individuals with compartmentalized virus has been shown to contribute to enhanced fusogenicity in HIV-1 *env* variants from patients with AIDS [97]. With the exception of the V1, C4 and C5 regions, multiple positions that discriminate CSF and plasma SGS populations in more than one studied individual can be found throughout the variable and constant regions of the receptor binding domain gp120 and the fusion protein subunit gp41 which work in concert to catalyze virus entry [98].

The evolution of amino acid sequences is naturally constrained by the need to maintain protein structure and function [99]. The interpretation of correlated mutational behavior (the tendency of amino acid positions in a protein to mutate coordinately) therefore allows for the inference of potential physical or functional interaction [100]. The identification of such correlated substitutions of amino acids has been applied in several instances to identify mutational clusters in HIV-1 reverse transcriptase and protease in response to ART [101–104] and to identify gp41 mutations that are significantly associated with particular HIV-1 V3 signatures that influence co-receptor usage [105]. We hypothesized that correlation analyses of amino acid positions in SGA-derived full-length HIV-1 *env* CSF variants would identify positions that share mutual information. In this study, novel correlated mutation analyses reveal that a subset of the amino acid residues identified in the initial compartmentalization hot spot positions form a network of significant correlations, with mutual information scores ≥ 0.10. We posit that these novel findings, made possible by the sequencing of full-length HIV-1 *env*, could provide potentially high-yield targets for downstream investigation of the implications of amino acid identity on the ability of the virus to persist in the CSF microenvironment, potentially via the infection of and enhanced replication in the various subtypes of CNS-resident macrophage and macrophage-type cells or microglia.

Structural mapping of the hot spot positions identified in this study onto the recently published SOSIP trimer structure revealed that these hot spots could be grouped into distinct spatial and functional clusters. This analysis suggested that these hot spot residues are located in regions potentially involved in trimer and CD4 binding site formation, and co-receptor binding. While entry of these viruses into the CSF via T-cells or as cell-free virus cannot be excluded, these data are consistent with the hypothesis that the CSF compartmentalized viruses are adapted for entry into CNS target cells and residency in the immune privileged CNS allows residues at these clusters to persist distinct from those in the plasma. For example, the V2 tip cluster is located at the apex center of the gp120, and alteration of these residues can destabilize the trimer, potentially allowing easier access to the CD4 bound conformation and the co-receptor binding site. Similarly, there are several residues in the hot spot cluster proximal to the CD4 binding site, and alteration of these residues may influence the formation of the CD4 binding site. This finding is of interest, as an enhanced ability for brain-derived HIV-1 *env* to use low levels of CD4 for virus entry in macrophages and microglia has been described [44]. Finally, structural mapping of the hot spot residues also supports some of the correlated mutation findings. For example, residues 362 and 392 linked in Figure 6B, are spatially next to each other in the cluster proximal to the CD4 binding site.

One limitation of the present study is the size of the study cohort. We sought to overcome this limitation by maximizing the amount and quality of the sequence data through the use of SGA. In this way we were able to identify sites where the dominant amino acid discriminates between CSF and plasma quasispecies within individuals using stringent statistical thresholds. The value of this method is seen in our ability to find both previously identified and novel compartmentalization sites across full-length HIV-1 *env*.

In an effort to identify specific HIV-1 *env* residues statistically associated with prevalent HAND in the study cohort, consensus sequences were derived from each individual’s CSF-variants. However, given the study size, it was not possible to report a statistically significant difference in a comparison of consensus sequences from those with NCN (n = 6) in comparison to those with ANI + MND (n = 9). Even at sites where the dominant residue differed completely between the two disease states, the maximum achievable fisher’s exact *p*-value of 2.0 × 10^-4^ would not fall under the Bonferroni corrected *p-*value threshold determined for comparison of variable sites across all 9 consensus sequences (*p <* 1.24 × 10-^4^). Larger studies, in which full-length HIV-1 *env* sequencing is performed in datasets that clearly discriminate between those with NCN and those with both ANI and MND disease is warranted, particularly as the investigation of individuals with weaker neurocognitive phenotypes may make uncovering genetic correlates of disease more difficult. We were also unable to identify viral genetic characteristics allowing us to discriminate between HIV-1 *env* quasispecies from individuals with NCN and those with ANI and MND. Mean viral diversity in both the CSF and plasma compartments did not differ significantly between individuals with NCN and those with ANI or MND and V1V2 loop length in CSF-derived variants CSF did not differ significantly between individuals with NCN and those with ANI or MND. The development of HAND is likely multifactorial, and the integration of knowledge about particular host social and genetic determinants of disease including HLA typing would be appreciated, but was not feasible within the confines of this study.

This study presents a cross-sectional analysis of a cohort of individuals with and without HAND. While highly informative, by definition this analysis captures a static view of the dynamic composite events that lead to the compartmentalization of virus, adaptation of viral variants to the CSF and the development of disease in those eventually diagnosed with HAND. As studies following individuals during primary infection have demonstrated, compartmentalization of virus to the CSF can be transient [59]. Additional studies following the specific genetic characteristics within CSF quasispecies from primary HIV-1 infection prior to the development of compartmentalization and/or HAND diagnosis are needed.

Finally, in interpreting these data, we must also consider the potential influence of the founder effect, particularly as it relates to the investigation of the described compartmentalization hot spots and the correlated network of residues at those sites. In an effort to report discriminatory changes that we believe are likely to result from evolutionary selection, we studied a group of unrelated chronically infected participants and restricted our reported findings to those common to a minimum of two unrelated individuals. The fact that other researchers using geographically and temporally distinct cohorts have described a subset of the sites reported in this study suggest that these changes in HIV-1 *env* may largely arise from distinct selective pressures.

## Conclusions

Detailed analyses of SGA-derived full length HIV-1 *env* from subjects with normal neurocognitive performance and those with the most common HAND diagnoses in the cART era allowed us to identify novel and previously described HIV-1 *env* genetic determinants of neuroadaptation and relate potential motifs to envelope structure and function. We demonstrated significant HIV-1 *env* compartmentalization between the CSF and plasma in a subset of individuals across neurocognitive disease states and cART status. In individuals with compartmentalized virus, mean HIV-1 *env* diversity was statistically lower in CSF- than in plasma-derived variants. In addition, mean V1V2 loop length was shorter and the mean number of PNLGS was lower in CSF-derived variants compared to their paired plasma counterparts, supporting the hypothesis that immune selection pressures are reduced in the privileged CNS compartment. Analysis of molecular patterns of compartmentalized HIV-1 *env* quasispecies revealed a series of compartment-discriminating positions (hot spots) within both variable and constant regions of HIV-1 *env* that are shared across non-related individuals, and a subset of amino acid residues within these hot-spots are statistically correlated to one another, suggesting a shared functional role. Structural mapping of the hot spot positions identified in this study suggests that these residues are located in regions potentially involved in trimer and CD4 binding site formation, and co-receptor binding. A combination of genetic features likely distinguishes viral populations compartmentalized to the CSF. The use of SGA and phylogenetic approaches is an effective method for identifying genetic features of neuroadaptation within the HIV-1 *env* gene and further experimental validation of predictions arising from these analyses will enable us to better understand the ways in which HIV-1 adapts to the CNS microenvironment.

## Methods

### Ethics statement

The CHARTER study was approved by the University of California, San Diego (UCSD) Human Research Protections Program (San Diego, California, United States). For all non-UCSD sites, the Human Research Protections Program at each enrolling site approved the research. All participants in the CHARTER study provided written informed consent prior to sample acquisition and all clinical investigation was conducted according to the principles expressed in the Declaration of Helsinki. The author’s use of de-identified clinical samples from the CHARTER study was approved by the Institutional Review Board of the Rockefeller University (New York, New York, United States).

### Study subjects and sample acquisition

Study subjects were chosen from the CHARTER cohort. De-identified, contemporaneous, cryopreserved CSF and plasma samples from fifteen chronically infected, HIV-1 seropositive individuals with normal neurocognitive performance (NCN), Asymptomatic Neurocognitive Impairment (ANI) and Mild Neurocognitive Disorder (MND) were provided.

Neurocognitive testing and clinical histories were obtained at CHARTER study visits by trained psychometrists and research staff. Participants underwent a comprehensive neurocognitive battery of tests within seven cognitive domains: speed of information processing, learning, recall, abstraction/executive functioning, verbal fluency, attention/working memory and motor skills. Following the demographic correction of T-scores for each test measure, a global deficit score (GDS), based on number and magnitude of impaired test performances was determined. At the time of neurocognitive testing, contemporaneous cerebrospinal fluid (CSF) and peripheral-blood samples were obtained from each individual by lumbar puncture and routine phlebotomy. Peripheral blood CD4+ T cell counts were performed at CHARTER research sites using routine established methods. HIV-1 RNA levels in the CSF and plasma were determined using the Roche Amplicor, version 1.5, with a lower limit of quantitation of 50 copies/mL.

### RNA extraction, generation of cDNA and single genome amplification

Thawed CSF and plasma samples were centrifuged at 2,500 rpm for 10 minutes to remove any contaminating cellular debris. HIV-1 in clinical samples was then concentrated by centrifugation for 2 hours at 25 K x *g.* Supernatant was removed down to 140 μL and the viral pellet resuspended. Viral RNA from clinical samples was extracted by routine methods using the QIAamp Viral RNA Mini Kit (QIAGEN, USA). To minimize the risk of within-patient cross contamination of samples, only one participant sample from one compartment (CSF or plasma) was processed on any given day.

We used published methods for SGA to generate cDNA and amplify single proviral molecules of full-length HIV-1 subtype B *env* gene (>2.5 kb) [55]. Reverse transcription (RT) of RNA to cDNA was performed using SuperScript III reverse transcriptase (Invitrogen Life Technologies, Carlsbad, CA). Briefly, 50 μL of RNA template, 0.5 mM deoxynucleoside triphosphates (dNTPs), 0.25uM primer *env*3out 5′- TTGCTACTTTGGATTGCTCCATGT-3′, and RNase-free water were incubated for 5 min at 65°C in a total volume of 65 μL to denature the secondary structure of the RNA. First-strand cDNA synthesis was carried out with 10 u/μL SuperScript III, 1x reverse transcriptase buffer, 2 u/μL RNase inhibitor (RNaseOUT, Invitrogen Life Technologies, Carlsbad, CA) and 5 mM DTT. Following reverse transcription, the reaction mixture was heat-inactivated followed by RNase H digestion (Invitrogen Life Technologies, Carlsbad, CA) at 37 degrees Celsius for 20 minutes. The resulting cDNA was used immediately for PCR or frozen at 80°C to await further analysis. All RNA extractions and amplification reactions were carried out with appropriate negative controls in parallel to detect contamination at each step of the procedure. cDNA was serially diluted and distributed in replicates of 10 PCR reactions in MicroAmp 96-well plates (Applied Biosystems, Foster City, CA) and cDNA was endpoint diluted in 96-well plates such that fewer than 30% of the PCRs yielded an amplification product. Additional PCR amplifications were performed using this dilution in 96-well reaction plates. PCR amplification was carried out in presence of 1x High Fidelity Platinum Taq PCR buffer, 2 mM MgSO4, 0.2 mM each deoxynucleoside triphosphate, 0.2 μM each primer, and 0.025 units/ μL of Platinum Taq High Fidelity polymerase in a 20 μL reaction (Invitrogen, Carlsbad, CA). The nested primers for generating full-length *env* were as follows: 1^st^ round sense primer *env*5out 5′-TAGAGCCCTGGAAGCATCCAGGGAAG-3′, 1^st^ round antisense primer *env*3out 5′- TTGGCTACTTGTGATTGCTCCATGT-3′, 2^nd^ round sense primer *env*5in 5′-TTAGGCATCTCCTATGGCAGGGAAGAAG-3′ and 2^nd^ round antisense primer *env*3in 5′-GTCTCGAGATACTGCTCCCACCC-3′. PCR parameters were as follows: 94°C for 2 min, followed by 35 cycles of 94°C for 15 s, 55°C for 30 s, and 68°C for 4 min followed by a final extension of 68°C for 15 min. The product of the first-round PCR was used as a template in the second-round PCR under the same conditions with the following PCR parameters: 94°C for 2 min, followed by 45 cycles of 94°C for 15 s, 55°C for 30 s, and 68°C for 4 min followed by a final extension of 68°C for 15 min. The resulting amplicons were then inspected on an E-Gel® 96 2% with SYBR® Safe (Invitrogen Life Technologies, Carlsbad, CA). All PCR procedures were carried out under clean PCR conditions with appropriate negative controls.

### DNA Sequencing

HIV-1 *env* gene products were directly sequenced using an automated ABI Prism 3730xl DNA analyzer (Applied Biosystems, Inc.). Both strands of DNA were sequenced with partially overlapping fragments. All sequencing chromatograms were carefully inspected for sites of ambiguous sequence (double peaks). Sequences for which any chromatogram revealed double peaks were excluded from further analysis, as this was indicative of amplification from more than one template or an early taq polymerase error.

### Sequence alignments

The CAP3 DNA sequence assembly program was used to concatenate sequence fragments for each pcr product [106]. Multiple alignments of nucleotide sequences were produced using Clustal W [107] with the following parameters: pairwise alignment gap opening penalty 10; gap extension penalty 0.1; multiple alignment gap opening penalty 10; gap extension penalty 0.2. Nucleotide or protein profile alignments were produced with ClustalX [107]. All resulting alignments were inspected and edited the Alignment Explorer in the MEGA 5.2 software when warranted [78]. Multiple alignments of codon and protein sequences were produced using Gene Cutter, a sequence alignment and protein extraction tool on the Los Alamos HIV database site http://www.hiv.lanl.gov. This algorithm first codon-aligns the input alignment, then translates the codon-alignment in frame using Hmmer v 2.32 with a training set of the full-length genome alignment.

### Phylogenetic tree construction and sequence diversity analysis

Nucleotide-based phylogenetic trees were constructed by the maximum likelihood (ML) method using the General Time Reversible plus Gamma (GTR + G) evolutionary model in the PhyML program [108]. Columns with gaps were removed from the multiple alignments using GapStrip/Squeeze v 2.1.0 with a gap tolerance of 50% on the Los Alamos HIV database site (http://www.hiv.lanl.gov) prior to constructing individual patient phylograms. Statistical evaluation of branch support in each phylogeny was performed using the approximate likelihood ratio test (aLRT) with SH-like supports [109]. The ProtTest tool was used to determine the most appropriate protein substitution model for data description. The “distance matrix” calculation in MEGA 5.2 was used to determine average pairwise genetic distances within or between compartments [78]**.** Overall, the phylogenetic model found to best describe the protein data while allowing for distance matrix calculations to be performed in MEGA 5.2 was the JTT plus Gamma model [110].

### Hypermutation analysis

Enrichment for mutations with APOBEC3G/F signatures was assessed using Hypermut 2.0 (www.hiv.lanl.gov) [111]. For each intra-patient sequence set, the most recent common ancestor (MRCA), a hypothetical viral sequence representing the most recent viral variant from which a subject’s viral quasispecies are descended was used as the reference sequence. MRCAs were reconstructed in DIVEIN using maximum likelihood methods [112]. Sequences that yielded a Fischer’s exact *p-*value of 0.05 or lower were considered significantly hypermutated and excluded from analyses of sequence diversity.

### Compartmentalization analysis

The Slatkin-Maddison test was used to detect population structure amongst HIV-1 *env* sequences within individual ML phylograms [62]. Implemented in the HyPhy software package [63], this approach applies a parsimony criterion to the evolution of each character on the maximum likelihood gene phylogeny in question, and assesses the degree of variation from the normal distribution of simulated sequences over the tree to assess the degree of intercompartment segregation. The significance of group separation was determined using the permutation test (10,000 permutations). Shifts in population structure were also calculated using a nonparameteric test for panmixia [60]. Derived from a geographic subdivision detection test proposed by Hudson et al. [61], this test compares an estimate of the degree of genetic differentiation in subpopulations of single genome sequences (SGS) chosen for comparison. The online version of this test was applied from the site at http://wwwabi.snv.jussieu.fr/~achaz/hudsontest.html. In the absence of genetic differentiation between subpopulations, random reassignment of SGSs to different groups would be expected to recapitulate a new, imaginary population with population structures with the same distribution as the experimentally observed subpopulation. Ten thousand (10,000) re-labelings/permutations were used to generate a *p-*value quantifying the statistical significance of the compartmentalization estimate.

### Coreceptor usage phenotype determination

V3 loop nucleotide sequences were extracted from multiply aligned full-length HIV-1 *env* for each participant using coordinates 7110-7216 on the HXB2 reference genome via the Gene cutter program on the HIV Los Alamos website www.hiv.lanl.gov. Translated V3 loop sequences were scored using Geno2Pheno [66] and the SINSI position-specific scoring matrix [PSSM] prediction algorithm [67].

### N-glycosylation determination

The N-Glycosite webserver [79] on the Los Alamos HIV database site (http://www.hiv.lanl.gov) was used to identify potential N-linked glycosylation sites (PNLGS) across HIV-1 *env* protein sequences.

### Compartmentalization signature pattern analysis

Signature pattern analysis was performed using the Viral Epidemiology Signature Analysis (VESPA) software [80] available on the Los Alamos HIV database site (http://www.hiv.lanl.gov). For each individual, the amino acid alignments of CSF-derived SGS (query) was compared to the amino acid alignments of contemporaneous plasma-derived SGS (background).

### Evaluation of the reliability of multiple sequence alignments

The web-based GUIDANCE program (http://guidance.tau.ac.il/overview.html) was used to construct a set of multiple sequence alignments and evaluate their reliability [81].

### Covariation analysis

Correlated mutations were detected using the CorMut package (Bioconductor version: release 2.12) for the R software environment for statistical computing and graphics [82]. Individual codon-aligned multiple alignments containing the reference plasma consensus sequence followed by all paired CSF-derived SGS were used as input. CorMut uses a mutual Information approach to detect correlated mutations. For each pair of positions in HXB2, a *p-*value was calculated by shuffling one of the alignment columns 10,000 times, calculating new random mutual information for each shuffled column and determining the fraction of random mutual informations that are greater or equal to the true mutual information. A mutual information (MI) score of 0.10 was chosen as a cut-off and as an additional criteria, mutations were considered significantly correlated if the Benjamini–Hochberg adjusted *p*-value for the correlation was less than 0.05 (corresponding to a 5% false discovery rate). The resulting adjacency matrix representing correlations between amino acids at investigated positions was constructed in CorMut.

### Mapping of compartmentalization hot spot positions to HIV-1 *env* trimer

To understand the spatial relation of the hot spot residues and their potential functional implication, we mapped these residues onto the recently published crystal structure of the SOSIP trimer (PDB ID 4NCO) [83]. To illustrate the CD4 binding site on the trimer, a crystal structure of gp120 core in complex with CD4 (PDB ID 1GC1) was superimposed with the SOSIP trimer and CD4 footprint was projected on the surface of the gp120 trimer. The structural figures were rendered with Pymol software package (http://www.pymol.org).

### Statistical analyses

With the exception of correlated mutations, all statistical analyses were performed using GraphPad Prism version 5.0d for Mac OS X, GraphPad Software, La Jolla California USA, www.graphpad.com.

### Availability of supporting data

All of the HIV-1 *env* sequences discussed in this manuscript have been deposited in GenBank (accession numbers KM258899 - KM259615).

## Additional files

Additional file 1: Figure S1. No significant correlation between differences in Amino Acid Diversities and Viral Load Between Compartments. Linear regression analysis comparing the ratio of each subject’s plasma to CSF average pairwise distance (APD) to the log of the ratio of the plasma to CSF HIV-1 RNA level (VL) is shown. The linear regression score (r^2^) was derived in PRISM. *P*-values <0.05 are considered significant. Additional file 2: Table S1. Bonferroni corrected *p-*value thresholds for compartmentalization analyses. The number of variable sites as determined in the MEGA 5.2 software in each subjects’ protein multiple-alignment was used to calculate the corrected Bonferroni *p-*value threshold. Each multiple-alignment was composed of the subjects CSF- and plasma-derived single genomes. #CSF SGS and #Plasma SGS = number of SGS used in analysis after exclusion of duplicate sequences within each compartment and sequences with statistical evidence of hypermutation. Additional file 3:
**Dataset S1.** The Complete list of Amino acids for each pair of sites exhibiting significant Mutual Information. Discrete amino acid positions from patient alignments are identified by their position in HXB2 gp160. Each site is identified by a nomenclature listing the dominant amino acid in the plasma consensus for the patient, followed by the HXB2 gp160 numbered position, concluding with the dominant amino acid in the CSF-derived sequences that represents a mutation from the dominant plasma consensus amino acid.
