# Supplementary figures and images for: Single genome analysis reveals genetic characteristics of Neuroadaptation across HIV-1 envelope
Source: Retrovirology. 2014 Aug 15;11:65. doi: 10.1186/s12977-014-0065-0 (PMC4145222; doi:10.1186/s12977-014-0065-0)

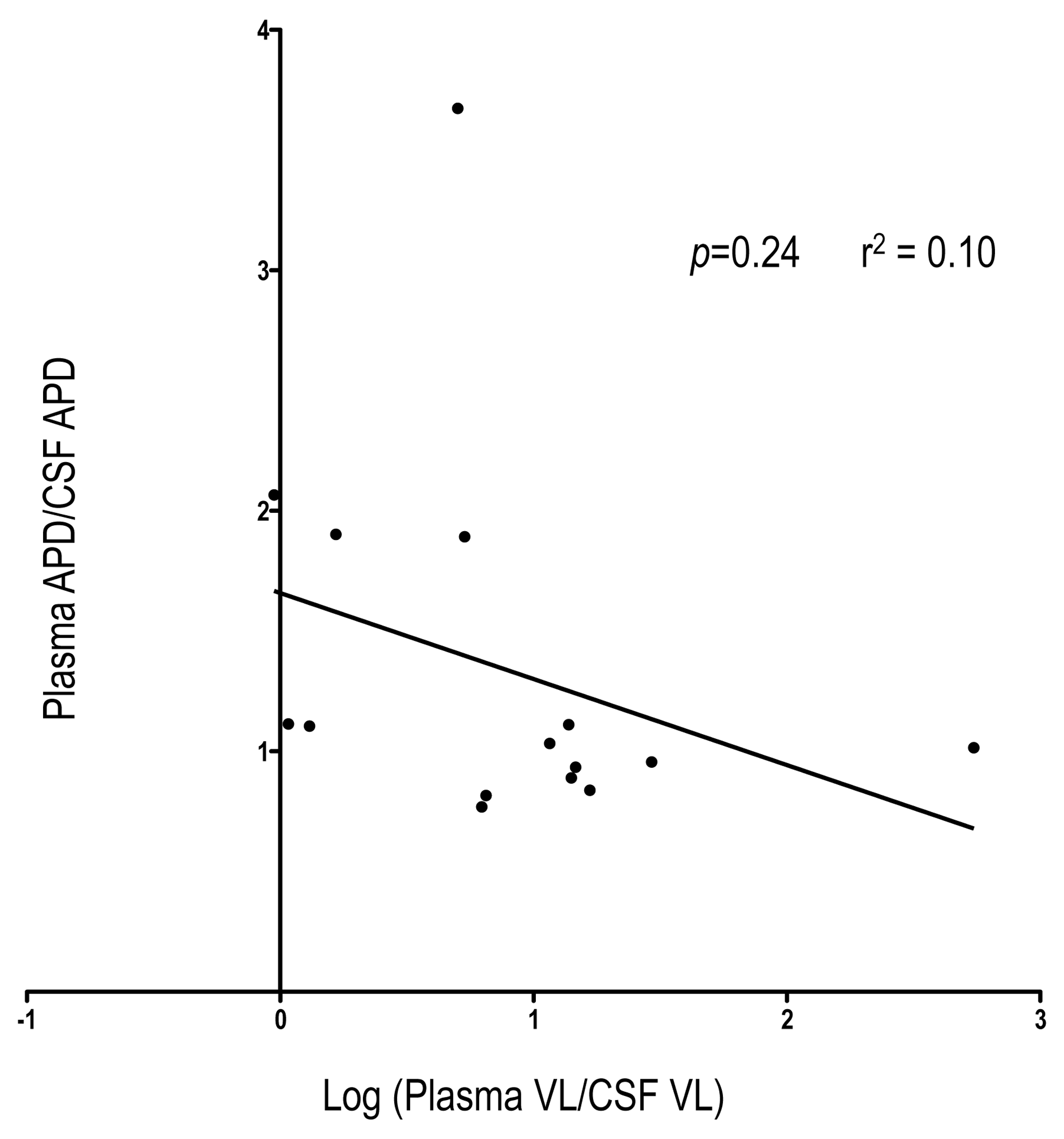

Supplement: Additional file 1: Figure S1. — No significant correlation between differences in Amino Acid Diversities and Viral Load Between Compartments. Linear regression analysis comparing the ratio of each subject’s plasma to CSF average pairwise distance (APD) to the log of the ratio of the plasma to CSF HIV-1 RNA level (VL) is shown. The linear regression score (r2) was derived in PRISM. P-values <0.05 are considered significant. [file 12977_2014_65_MOESM1_ESM.tiff]
