# Supplementary material for: Single genome analysis reveals genetic characteristics of Neuroadaptation across HIV-1 envelope
Source: Retrovirology. 2014 Aug 15;11:65. doi: 10.1186/s12977-014-0065-0 (PMC4145222; doi:10.1186/s12977-014-0065-0)
Supplement: Additional file 2: Table S1. — Bonferroni corrected p-value thresholds for compartmentalization analyses. The number of variable sites as determined in the MEGA 5.2 software in each subjects’ protein multiple-alignment was used to calculate the corrected Bonferroni p-value threshold. Each multiple-alignment was composed of the subjects CSF- and plasma-derived single genomes. #CSF SGS and #Plasma SGS = number of SGS used in analysis after exclusion of duplicate sequences within each compartment and sequences with statistical evidence of hypermutation. [file 12977_2014_65_MOESM2_ESM.docx]

| **Subject** | **# CSF SGS** | **# Plasma SGS** | **Number of Variable Sites** | **Bonferroni *p-*value threshold** |
| --- | --- | --- | --- | --- |
| NCN1 | 6 | 20 | 72 | 0.0007 |
| NCN2 | 21 | 22 | 160 | 0.0003 |
| NCN3 | 21 | 26 | 100 | 0.0005 |
| NCN4 | 34 | 19 | 209 | 0.0002 |
| NCN5 | 26 | 19 | 193 | 0.0003 |
| NCN6 | 18 | 15 | 162 | 0.0003 |
| ANI1 | 25 | 25 | 186 | 0.0003 |
| ANI2 | 19 | 21 | 217 | 0.0002 |
| ANI3 | 12 | 17 | 146 | 0.0003 |
| ANI4 | 22 | 36 | 148 | 0.0003 |
| ANI5 | 20 | 19 | 88 | 0.0006 |
| ANI6 | 26 | 28 | 108 | 0.0005 |
| ANI7 | 24 | 23 | 184 | 0.0003 |
| MND1 | 20 | 26 | 134 | 0.0004 |
| MND2 | 16 | 29 | 108 | 0.0005 |
